# Lowering maintenance immune suppression in elderly kidney transplant recipients; connecting the immunological and clinical dots

**DOI:** 10.3389/fmed.2023.1215167

**Published:** 2023-07-12

**Authors:** Michiel G. H. Betjes, Annelies De Weerd

**Affiliations:** Department of Internal Medicine, Erasmus MC Transplant Institute, University Medical Center Rotterdam, Rotterdam, Netherlands

**Keywords:** T cell-mediated rejection, kidney transplantation, graft survival, age, mortality, antibody-mediated rejection, donor-specific antibodies, immune suppression

## Abstract

The management of long-term immune suppressive medication in kidney transplant recipients is a poorly explored field in the area of transplant medicine. In particular, older recipients are at an increased risk for side effects and have an exponentially increased risk of infection-related death. In contrast, an aged immune system decreases the risk of acute T-cell-mediated rejection in older recipients. Recent advances in alloimmunity research have shown a rapid and substantial decline in polyfunctional, high-risk CD4^+^ T cells post-transplantation. This lowers the direct alloreactivity responsible for T-cell-mediated rejection, also known as donor-specific hyporesponsiveness. Chronic antibody-mediated rejection (c-aABMR) is the most frequent cause of kidney graft loss in the long term. However, in older adults, c-aABMR as a cause of graft loss is outnumbered by death with a functioning graft. In addition, DSA development and a diagnosis of c-aABMR plateau ~10 years after transplantation, resulting in a very low risk for rejection thereafter. The intensity of immune suppression regimes could likely be reduced accordingly, but trials in this area are scarce. Tacrolimus monotherapy for 1 year after transplantation seems feasible in older kidney transplant recipients with standard immunological risk, showing the expected benefits of fewer infections and better vaccination responses.

## Highlights

- The increasing number of older patients who have undergone kidney transplants in the recent decade is likely to increase further.
- The aging of the adaptive immune system lowers the risk of rejection after kidney transplantation.
- Immunosuppressive drugs have more side effects in older adults and increase the risk of de novo diabetes mellitus and serious infections.
- After kidney transplantation, the frequency of risky polyfunctional alloreactive CD4 T cells declines through activation-induced apoptosis, leading to donor-specific hyporesponsiveness.
- By integrating insights into immunological aging, the appearance of donor-specific hyporesponsiveness, and data from trials on lowering immune suppression, it is possible to outline a rationale for diminishing immune suppression intensity in older recipients after the early months of transplantation and to promote living kidney donation.

## Introduction

Over the recent decades, significant progress has been made regarding kidney allograft survival in the first year after transplantation by optimizing immune suppression. In parallel, the number of kidney transplantations performed in elderly ESRD patients has increased due to improved life expectancy (1, 2). The proportion of transplant candidates of 65 years and older continues to rise (2), and in the Netherlands, for example, the number of kidney transplant recipients aged 65 years and above increased between 2006 and 2021 from 1,181 (18% of the total number) to 4,384 (36% of the total number), and for recipients aged 75 years and above, an even more striking increase from 163 to 1,319 was noted (source: www.nefrovisie.nl/nefrodata). This increase in older kidney transplant recipients has led to new, largely unanswered questions about what should be the optimal treatment regimen with immune suppressive drugs.

In contemporary times, most immune-suppressive regimens consist of induction with an Il-2R blocking monoclonal antibody (basiliximab) or T-cell depletion (ATG or alemtuzumab), followed by triple immune suppression. The maintenance of immune suppression in the vast majority of patients consists of tacrolimus, mycophenolate mofetil (MMF), and steroids. Using this regimen, the allograft survival of kidneys from living donors at 1 year is >98% in most studies (3). The 1-year graft survival of deceased donor kidney allografts is usually >90% but varies with the quality of the accepted organ, which is determined by the donor's age, co-morbidity of the donor (e.g., hypertension, diabetes), type of donation (brain death or cardiac-death donation), and cold ischemia time (4–6). The risk for acute rejection, which is predominantly T cell-mediated, is highest in the first weeks after transplantation and decreases thereafter (7, 8). After 3–5 years, the occurrence of acute rejection is virtually non-existent in compliant patients; however, it can still occur if an immune suppressive medication, particularly tacrolimus, is significantly lowered or discontinued (7–9). The time-dependent phenomenon is rooted in the immunological concept known as donor-specific hyporesponsiveness (DSH), indicating a substantial decline in T cell-mediated donor-specific immune reactivity (10–12). However, while the risk for acute rejection has become negligible several years after transplantation, the cumulative risk for chronic allograft rejection increases (13, 14). This type of rejection is predominantly caused by chronic-active antibody-mediated rejection (c-aABMR), which is now recognized as the major cause of graft failure (8, 15). The second most frequent cause of long-term graft loss is chronic damage reflected by interstitial fibrosis and tubular atrophy (IFTA) in biopsies. This may partly be mediated by ongoing TCMR (iFITA) or by the nephrotoxicity of tacrolimus (8, 16–18).

A hitherto unanswered question is how long-term immune suppressive medication should be adapted in light of the occurrence of DSH, on the one hand, and c-aABMR and IFTA, on the other hand (19). To make the discussion even more complex, using immune suppressive drugs poses an increased risk for malignancies, infections, and cardiovascular disease. In this discussion, the age of the recipient is of pivotal importance, as the risk of rejection decreases with age while the risk of infection increases, and mortality becomes an important competing risk factor in graft survival (8).

In this review, we discusse the different immunological and clinical parameters that can guide immune suppressive medication in the long term, with an emphasis on older kidney transplant recipients.

## The aging immune system and kidney transplantation

**Figure 1:**
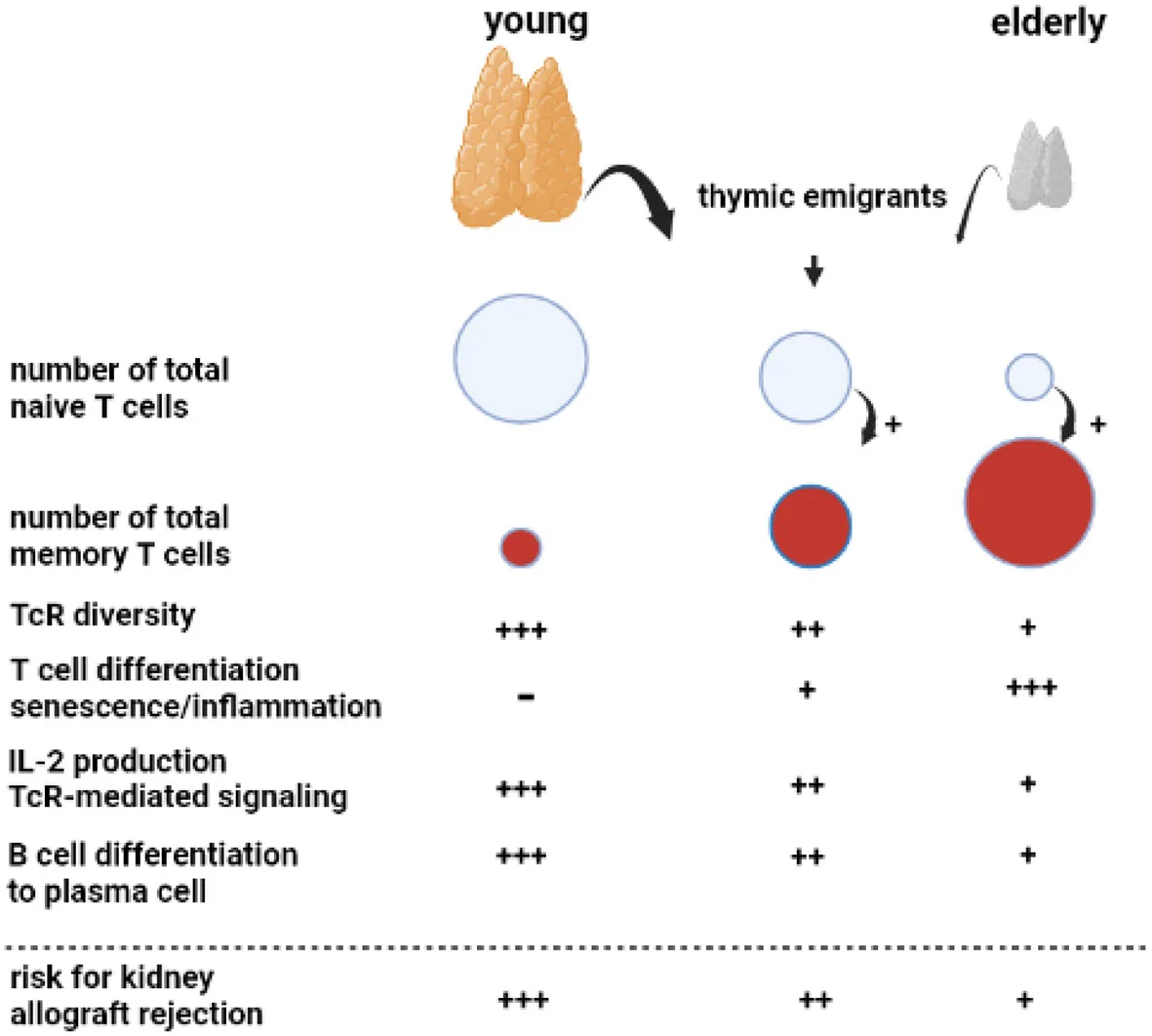
Aging of the adaptive immune system. A key phenomenon with aging is progressive atrophy of the thymus, called thymus involution, which sharply reduces the number of newly formed naïve T cells released as thymic emigrants in the circulation. Repetitive encounters with a diversity of pathogens shape and increase the total memory T cell population. This memory T-cell inflation is enhanced in the presence of decreased numbers of naïve T cells, as observed in the elderly. The decreasing output of thymic emigrants and expanding memory T-cell population decrease T-cell receptor (TcR) diversity. Memory T-cell inflammation is associated with increasing numbers of highly differentiated T cells, which can become senescent and have a proinflammatory profile, most prominent in relation to cytomegalovirus infection. In particular, CD4 T cells in older adults have shown less Il-2 production because of impaired downstream TcR-mediated signaling. The B cell compartment is diffusely negatively affected by age with the impaired germinal center formation in the lymphnode, leading to a less vigorous antibody response. Of note, all age-related changes in T and B cells are increased by renal failure, leading to premature immunological aging by an average of 15–20 years.

The adaptive immune system consists of T and B cells, which, in transplantation immunology, are of pivotal importance as they are the main drivers of cellular and antibody-mediated rejection. Increasing age has a clear negative effect on both T and B cell numbers and function (Figure 1) and antibody development. End-stage kidney disease aggravates these processes in older kidney transplant recipients.

An important phenomenon is progressive age-related atrophy of the thymus, which leads to declining production of newly formed naïve T cells that enter the circulation as recent thymic emigrants (20, 21). The consequence is an almost linear decrease in the circulating pool of recent thymic emigrants' T cells with increasing age, alongside a concomitant decrease in T cell receptor diversity (22, 23). The number of naïve CD4^+^ T cells decreases relatively little because of homeostatic proliferation, while the number of circulating naïve CD8^+^ T cells becomes very low in older adults (21, 23, 24). Memory T cells, which have differentiated and expanded from naïve T cell precursor cells after antigenic stimulation, are increasing with age due to repetitive antigen encounters, e.g., via virus and bacterial infections. In addition, the decrease in naïve T cells *per se* favors memory T-cell expansion (20). The age-dependent expanding pool of circulating memory T cells shows increased signs of senescence and lower expression of the costimulatory molecule CD28 (25) and is believed to contribute to low-grade inflammation often found in older adults (hence the term inflamm-aging) (26). In particular, latency for cytomegalovirus (CMV) may lead to a large expansion of highly differentiated T cells in older adults and an increase in low-grade inflammation (27). The CD4^+^ T cells, as helper T cells important for cytotoxic CD8^+^ T cells and for antibody production, are also functionally different and produce less interleukin-2 with impaired signaling via the T cell receptor to the downstream intracellular p-ERK/p38 pathway (28).

Different stages of chronic kidney disease are associated with a progressive decline in naive T cells, which appears to be mediated by increased apoptosis and a decreased output of thymic emigrants (20, 29). All other aspects of an aging immune system, such as increased memory T-cell differentiation and skewing of the T cell receptor (TcR) repertoire, pronounced expansion of terminally differentiated CD4^+^ and CD8^+^ T cells in relation to CMV latency, and downregulation of the p-ERK/p38 pathway have been observed in chronic kidney disease (30–33). Taken together, patients with end-stage renal disease have a prematurely aged T-cell system by, on average, 15–20 years throughout all age categories (34). Unfortunately, no reversal of premature T cell aging is observed after kidney transplantation (35).

Age-related changes to circulating B cells lead to diminished immunoglobulin production and clonal expansion (36). In addition, kidney failure is associated with a diffuse loss of all circulating B cell subsets (37–39). Recent studies have indicated that germinal center formation, the dedicated areas in lymph nodes for the generation of memory B cells and plasma cells, is also impaired in older adults (40). The age-related changes in both T and B cells favor a less vigorous response to, in particular, new antigens. This relatively weak immune stimulation has also been observed with different vaccinations (41, 42). For instance, vaccination against hepatitis B with HBs antigen yields a lower serological response in older adults, particularly older patients with renal failure, and can be attributed to a much lower generation of Il-2-producing effector-memory T cells (43, 44). Of note, older adults can still mount a sufficient immune response to more potent vaccines such as an mRNA-based COVID vaccine and control infections to which memory-based adaptive immune responses have been formed in the past (45).

For an older kidney transplant recipient, the aging immune system is a potential threat because of the increase in memory T cells, which could lead to higher numbers of T cells cross-reactive with allogeneic HLA molecules. Although severe acute rejection may be observed in an older recipient, this is not the rule, and T-cell-mediated rejection is observed less frequently in an older recipient (see below). This indicates that the impaired functionality of aged T cells carries more weight and decreases rejection risk. This concerns, in particular, CD4^+^ T cells, which have an important role in both cellular and antibody-mediated rejection (AMR; see below), as the generation of *de novo* donor-specific antibodies leading to (chronic) antibody-mediated rejection will be less likely. The other side of the coin is the increased susceptibility of an older recipient to infection and a decrease in anti-tumor immune surveillance as immune suppression further weakens these lines of defense executed by T cells (24).

## The immunology of graft rejection: direct, semi-direct, and indirect T-cell alloreactivity

### The direct pathway of alloreactivity

**Figure 2:**
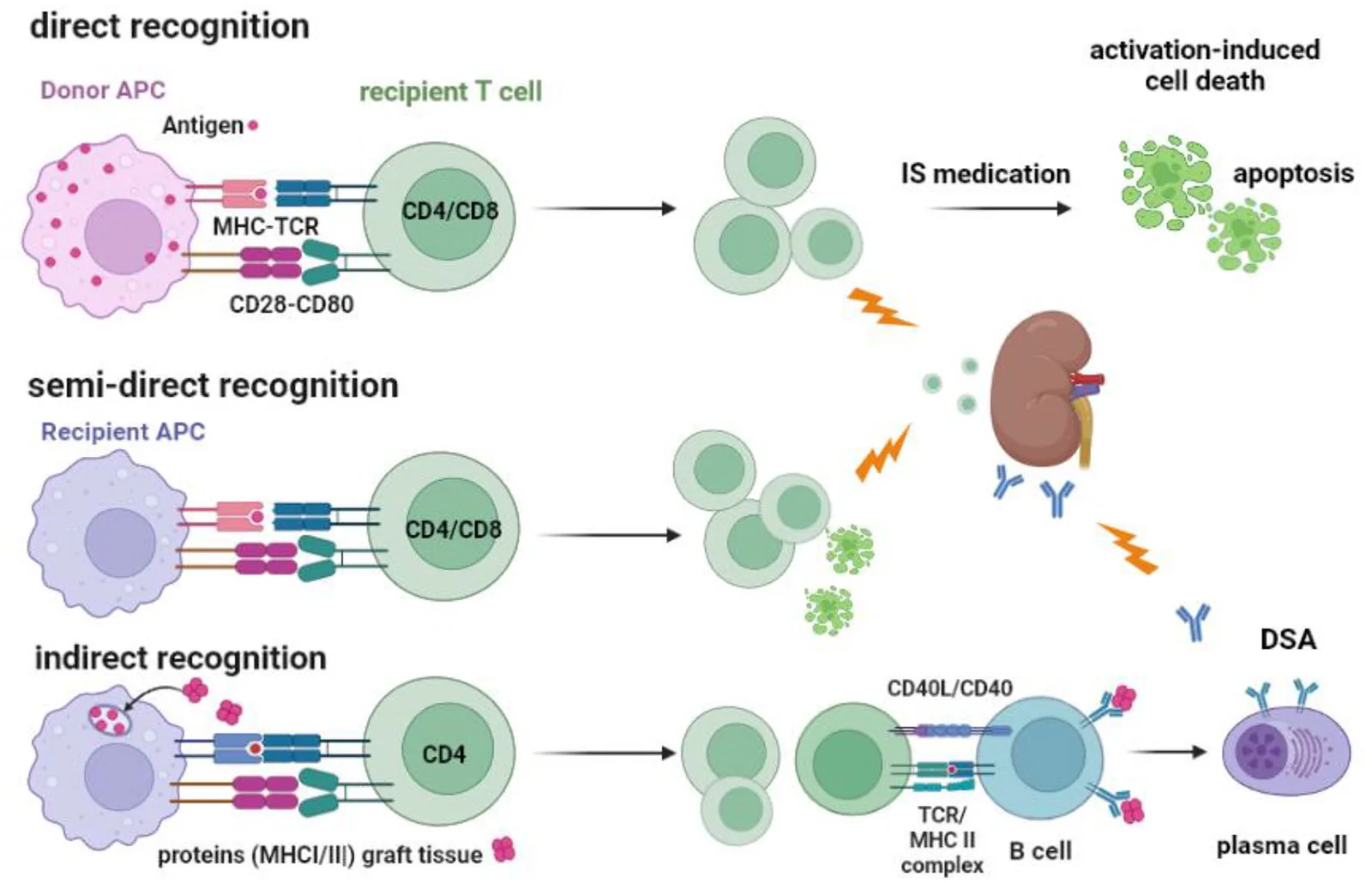
Recognition of allogeneic MHC by the recipients' T cells and the routes to cellular and antibody-mediated rejection. T cells can be activated via their T cell receptor by allogeneic MHC molecules (MHC-TCR) expressed on antigen-presenting cells (APC). Co-stimulation by, for example, CD28-CD80 interaction is an important second signal needed for full T-cell activation and proliferation. Direct recognition is the activation of the recipient T cell by the donor APC. Recipient APC within the transplanted kidney can take MHC molecules from donor cells and insert them into their cell membrane (“crossdressing”). Through this mechanism, the recipient APC can present allogeneic MHC to recipient T cells, which is called semi-direct recognition. Both CD4 and CD8 T cells can be activated by the direct and semi-direct pathways and cause acute T cell-mediated rejection, as indicated by the lightning arrow. Under the influence of immune suppressive (IS) medication, the activated T cells cannot be fully activated and may undergo apoptosis via a process called activation-induced cell death, which, in time, reduces the number of alloreactive T cells. The indirect pathway of recognition involves the classical route of antigen (e.g., donor MHC) taken up by the recipient APC and, after processing into an immunogenic peptide, presented in the context of MHC II to the recipient CD4 T cells. Antigen-specific CD4 T cells can interact with B cells to facilitate the transition to plasma cells producing donor-specific antibodies (DSA), a process that needs co-stimulation by CD40-CD40L interaction. DSA can cause antibody-mediated rejection, as indicated by the lightning arrow.

**Figure 3:**
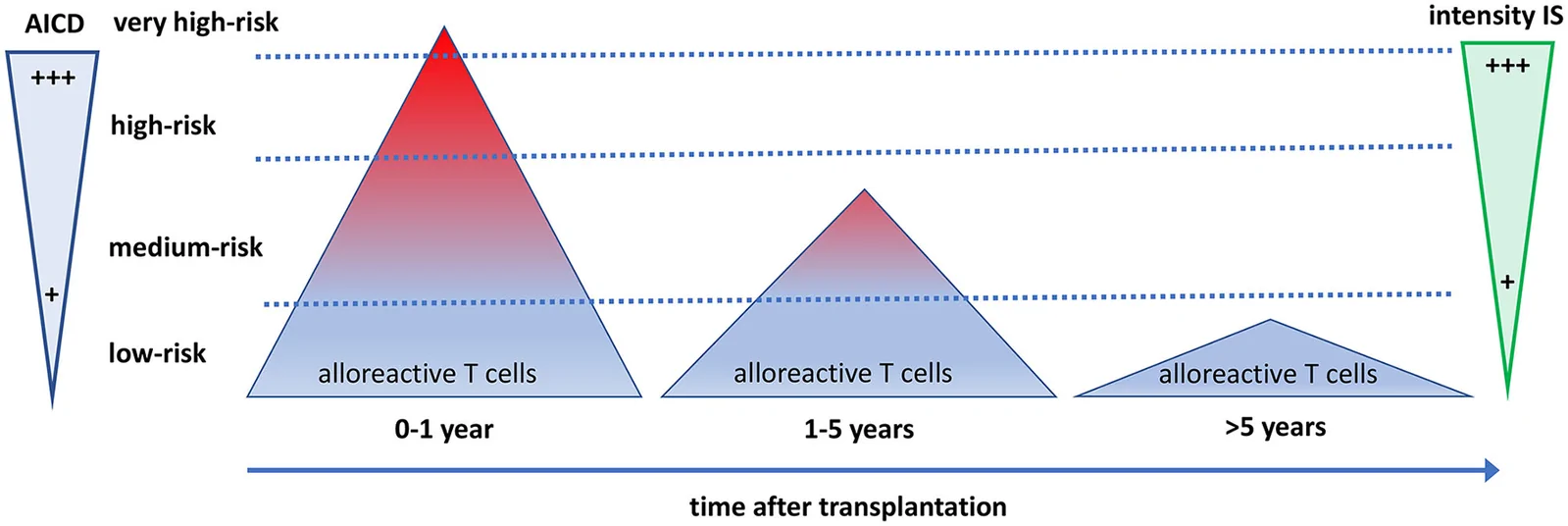
The quantity and quality of alloreactive T cells after transplantation in relation to the need for immune suppressive drugs and activation-induced cell death. The direct alloreactive T cell population is large and comprises 5–10% of the pool of total circulating T cells (triangles). The vast majority of alloreactive T cells do not express activation markers upon interaction with the allogeneic HLA (depicted as blue), and <1% express cytokines. The most potent alloreactive T cells express multiple cytokines (polyfunctional cells, depicted as red), yielding an increased risk for graft rejection. The more an alloreactive T cell can be activated by allogeneic HLA, the more it is prone to activation-induced cell death (AICD), causing a decline and eventually a total disappearance of polyfunctional high-risk alloreactive T cells post-transplantation. Different intensities of immune suppressive medication are needed to control the alloreactive T cells at different time periods after transplantation. Some highly potent alloreactive T cells escape immune suppression and AICD early after transplantation and will cause rejection of the graft (the little red triangle above the upper dotted line). On average, after 5 years, only low-risk alloreactive T cells have remained, which can be controlled by low-intensity immune suppression.

The initial risk for acute TCMR is believed primarily to be mediated by “direct T cell alloreactivity” (Figure 2), in which recipient T cells can be directly activated after engaging their TCR with the allogeneic donor HLA (46). Circulating T cells are “educated” in the thymus to respond primarily to immunogenic peptides in the context of self-HLA molecules (“self-restricted antigen presentation”). Allogeneic donor HLA molecules are by definition not part of this education and may interact with the recipient T cell receptor if there is a good-enough fit with the allogeneic HLA molecule (47–49). It is estimated that roughly 5–10% of the T cells are capable of this allo-recognition. This is a huge precursor frequency for immune reactivity, as frequencies of antigen-specific T cells are usually well below 0.1%. For example, post-hepatitis B (HBV) vaccination, HBV-specific T cells comprise <0.01% of total circulating T cells (43, 44, 50). However, only a fraction of the alloreactive T cells (~1 %) can be considered high-risk cells as they are sufficiently activated to produce cytokines. These cells can cause rejection of the allograft, even in the presence of immune suppressive medication (Figures 2, 3).

### The semi-direct pathway of alloreactivity

Recent studies have suggested that recipient antigen-presenting cells (APC) can take up allogeneic donor HLA molecules in their cell membranes (called “HLA crossdressing”), thereby contributing to the direct alloreactive T cell response (the “semi-direct route”) (51).

Given the rapid onset of most vascular rejections within a week, it is likely that only the recipients' memory T-cell subset is involved in causing this type of rejection (52, 53). These memory T cells can be rapidly activated by re-exposure to the specific antigen (the “recall” reaction) or, in the case of direct or semi-direct alloreactivity, a cross-reaction with allogeneic HLA. The alloreactive memory T cells may not need stimulation by APC and can be activated directly by HLA-expressing donor endothelial cells and proliferate upon IL-15 produced by parenchymal cells (53–55). Naive T cells that have not been antigen-activated (also called antigen-inexperienced) need to be switched on by professional antigen-presenting cells such as dendritic cells (56). This is a primary immune response for which naive T cells need to be activated in lymphoid tissue, followed by a transition to a memory type of T cell. Activation results in an expanded population of T cells with the capacity to produce inflammatory cytokines and display cytotoxicity to their target cells, in the case of CD8^+^ T cells. Of note, post-T cell priming rejection responses can be modified by targeting pathways that regulate T-cell trafficking, survival cytokines, or innate immune activation, as recently reviewed in detail (57).

### The indirect pathway of alloreactivity and the formation of donor-specific antibodies

The third important route of graft rejection is called indirect alloreactivity (Figure 2). Despite the misleading terminology, it actually involves the normal route of eliciting an antigen-specific T cell response via TCR-mediated recognition of an immunogenic peptide in the context of self-HLA by a T cell. This self-restricted antigen presentation is similar to the T cell response evoked by a viral infection or vaccination with a protein (43, 44). In the case of transplantation, the immunogenic peptides are derived from donor-derived proteins processed by recipients' APC and presented to recipient T cells in the secondary lymphoid organs. Importantly, this will also trigger a humoral immune response leading to plasma cells producing anti-donor-specific antibodies. While all donor-derived proteins theoretically can produce an antibody response, donor HLA molecules are the most immunogenic. Reliable, sensitive Luminex-based assays are now available to measure anti-donor HLA antibodies, which have supplemented the classical complement-dependent cytotoxicity (CDC) test (58). Kidney transplantation in the presence of anti-HLA DSA is possible but carries a higher risk of humoral rejection and increased graft loss even after desensitization prior to transplantation. The risk is highest for transplantation with a positive CDC test (59, 60).

Immune responses against non-HLA molecules should not be dismissed as irrelevant. For instance, the load of molecular mismatches between the donor and the recipient is associated with long-term graft loss, and antibodies to non-HLA molecules (e.g., anti-ATR1, anti-MICA, anti-ARGHDIB antibodies) may lead to ABMR and are associated with fibrosis (61–65). In addition, the polymorphism in Fc-receptor expression levels may modulate the clinical effect of circulating antibodies to the graft (66).

## Donor-specific hyporesponsiveness after kidney transplantation

### Loss of direct alloreactive T cells post-transplantation

The concept of donor-specific hyporesponsiveness **(**DSH) has long been recognized by clinicians based on clinical experience and experimental data. The risk for acute rejection, especially for the vascular type of T cell-mediated rejection, is highest in the first weeks after transplantation, after which the incidence of TCMR decreases rapidly (7, 8). Most post-transplantation immune suppressive protocols incorporate this ‘clinical knowledge' by lowering maintenance immune suppression after several months, for example, by steroid tapering and lowering target tacrolimus trough levels. Note that the incidence of early acute humoral rejection is low (<5% within the first year) unless donor-specific antibodies, mostly anti-HLA, were already present before transplantation (7, 67, 68).

The initial experimental data showing DSH were largely based on the proliferation of recipient T cells to donor APC *in vitro* by an assay called mixed leucocyte reaction (10). This assay measures the direct alloreactive T cell response and generally shows a strong proliferative anti-donor T-cell response *in vitro*, with decreasing proliferation in the years after transplantation. Several mechanisms may contribute to the occurrence of DSH, such as the anergy of alloreactive T cells, the regulatory effects of Tregs, and cell deletion (69). Studies in this field have shown varying results, with little evidence for the regulation of alloreactivity by Tregs (70, 71). A major drawback of these studies was that alloreactive T cells were not studied on a single-cell level. By using activation-induced markers such as CD154 or CD137, it is possible to identify alloreactive T cells through short-term culture in an MLR and subsequently conduct multiparameter cell analysis using flow cytometry or highly-selective alloreactive T-cell enrichment through cell sorting (43, 72–74).

Recent publications have shown by single-cell flow cytometry that the number of alloreactive T cells declines over time, particularly highlighting a decline in polyfunctional CD4^+^ T cells (those capable of producing two or more cytokines) due to activation-induced apoptosis (AICD) (69). Polyfunctionality identifies, in general, the T cells that have superior activity in driving an immunological process to an optimal cytotoxic or serological response (75–77). The decline of polyfunctional alloreactive CD4^+^ T cells could be measured 6 months after transplantation, indicating a relatively rapid loss of these high-risk alloreactive T cells. In addition, the pre-transplant frequency of polyfunctional alloreactive CD4^+^ T cells was shown to be associated with the risk of acute TCMR after kidney transplantation (78).

The process of AICD is a well-known immunological mechanism that uses the Fas-FasL apoptosis pathway to maintain T cell homeostasis and restrict the excessive proliferation of activated T cells (79). It seems likely that the administration of immune suppressive drugs impedes stimulation, such as IL-2 signaling, thereby inhibiting further activation of alloreactive T cells and facilitating AICD (80). Some highly activated alloreactive T cells may escape immune suppression (e.g., by using IL-15 as a growth factor) and will not be eliminated through AICD, causing graft rejection (Figure 3).

The decreased alloreactive proliferative T cell response in the MLR, which is the classical definition of DSH (12), is highly associated with the decline of polyfunctional alloreactive CD4^+^ T cells (69). Of note, alloreactive CD8^+^ T cells, as opposed to CD4^+^, remained largely unchanged for many years in numbers and gene expression profiles. However, after an average of 10 years after transplantation, alloreactive CD8^+^ T cells also start to decrease (Figure 3) (69).

These results showed that DSH is mediated primarily by the loss of alloreactive CD4^+^ T cells and, as such, are in accordance with experimental animal studies, which indicate a pivotal role of CD4^+^ T cells in allograft rejection (81–83). Moreover, recent studies have shown that a load of peptides that can be presented indirectly by the recipient HLA class II molecules (PIRCHE score) correlates with the risk for TCMR (84, 85). These data suggest that the indirect pathway involving CD4^+^ T cells may be more important in TCMR than previously believed.

Operational tolerance to the kidney allograft (no rejection after stopping all immune suppressive drugs) is a rare occasion (86). However, some kidney transplant recipients are clinically stable several decades after transplantation with (very) low doses of immune suppressive drugs. This is in line with our findings that frequencies of CD4^+^ alloreactive T cells persist for years, and frequencies of alloreactive CD8^+^ T cells take more than a decade to decrease substantially. Thus, T-cell alloreactivity becomes very low as the high-risk alloreactive T cells have virtually disappeared (Figure 3). In contrast to kidney transplantation, liver transplantation can lead to operational tolerance in a substantial number of recipients. However, a decrease in functionally alloreactive CD4 T cells has also been shown in operationally tolerant liver transplant recipients, which may indicate a similar underlying mechanism (87).

### The indirect alloreactive pathway and development of post-transplantation donor-specific antibodies

As the most potent part of the direct pathway of alloreactivity disappears over time, the indirect pathway is operative from the start of transplantation but probably at a slow pace and involves the activation of naive T cells, eliciting *de novo* immune responses. The indirect pathway leads to a low and fairly constant yearly incidence of ABMR of approximately 1.1% per year (8). Measuring indirect alloreactive T cells is notoriously difficult, as precursor frequencies are much lower than the direct response, and the assays are technically challenging (88). However, *de novo* DSA arising after transplantation is formed via this pathway and can be measured by sensitive Luminex-based assays. Most of the studies have shown a cumulative increase in the percentage of transplant patients who develop *de novo* DSA in the initial years after transplantation. The cumulative incidence appears to plateau after 5–10 years, along with the risk for ABMR due to *de novo* DSA (89, 90). This corresponds with the clinical data that, long after transplantation (>10–15 years), recipients rarely have a newly diagnosed ABMR. These recipients have proven that their HLA mismatches do not elicit a relevant indirect immune response under standard immune suppression. It is tempting to speculate that AICD of donor-HLA antigen-specific T cells also underlies this phenomenon and that some T cells become activated enough to circumvent immune suppression and AICD, while the majority of these cells will eventually disappear. However, no data are available to support this hypothesis.

As mentioned, not all transplanted organs elicit a similar alloreactive cellular and humoral immune response. There is a higher prevalence of operational tolerance in the liver than in kidney transplant recipients, although *de novo* DSA formation is associated with an increased risk for liver rejection and graft failure (91). The relative insensitivity of the liver graft to pre-existing DSA as opposed to the kidney graft can be explained by the unique expression of HLA-DR and DQ on renal endothelial cells (92). Moreover, in the kidney allograft, the inflammation caused by DSA is observed within the microvasculature, which is the glomerular and peritubular capillaries, whereas the arterioles frequently remain unaffected (93). This difference indicates that endothelial and possibly also parenchymal cells protect themselves against damaging inflammatory sequelae after interaction with DSA according to organ type and vasculature tissue within that organ. How this is mediated is currently unknown, but upregulation of cell-bound complement regulatory systems or expression of ligands involved in apoptosis (inducing cell death in activated T cells) are possible mechanisms (94, 95).

In conclusion, donor-specific hyporesponsiveness by activation-induced cell death of high-risk polyfunctional, direct alloreactive T cells is a potent mechanism by which the risk for TCMR is substantially lowered in the first year after transplantation, which continues thereafter. In contrast, the cumulative risk for c-aABMR caused by *de novo* DSA formation via the indirect pathway of alloreactivity increases and plateaus approximately 10 years posttransplant.

## The intensity of maintenance immune suppression regimens—Balancing between donor-specific hyporesponsiveness and ongoing indirect alloreactivity

Given the development of DSH by the progressive loss of high-risk polyfunctional alloreactive T cells, it is reasonable to consider lowering the immune suppressive drug load in the long-term management of kidney transplant recipients. Two important questions are as follows: Who can be considered for safe lowering? Which drugs should be considered? First, known risk factors for TCMR can be identified in the donor and the recipient. Donor-related factors such as delayed graft function, cold ischemia time, and deceased donor vs. living status mediate their effect by increasing the immunogenicity of the donor's kidney, as these factors lead to the upregulation of HLA and adhesion molecules (96). Furthermore, kidneys from older donors are more immunogenic, which is particularly relevant for younger recipients (97, 98). The total number of HLA mismatches is an important risk factor, as is the older age of the recipient, which confers a substantially lowered risk for TCMR, given the effects of an aging immune system (25, 31, 99–101). The initial trough level of tacrolimus, medication adherence, and type of induction therapy (none, CD25-blocking antibody, T cell depletion) are also modifiable risk factors for TCMR (102, 103). However, although these factors are important for initial TCMR risk assessment, they are not known to be associated with the long-term risk of TCMR, as this type of rejection essentially disappears after 3–5 years.

Many centers continue a maintenance triple immune suppressive drug regimen (usually tacrolimus, MMF, and prednisone), while others withdraw steroids at some point. Early steroid withdrawal (within 6 months after transplantation) increases the risk of TCMR by 10–20%, but long-term graft survival is not affected (104). Long-term continuation of prednisone may thus be superfluous in the prevention of TCMR. However, studies on steroid withdrawal many years after transplantation are lacking. This must be weighed against the apparent disadvantages of steroid use, such as hypertension, DM, osteoporosis, and infection. Steroid withdrawal has been proven beneficial as it lowers the risk of post-transplant DM, bone loss, and infections (104–110).

MMF has replaced azathioprine in current immune suppressive regimens. MMF use may cause serious gastrointestinal side effects and severely lower the antibody response to vaccination, e.g., COVID-19 vaccines (111–113). In general, the use of MMF is associated with more infections, particularly viral infections, but it does not seem to increase the risk for cancer significantly and may even have therapeutic potential as an anticancer drug (114–116). Thus, the potential benefits of steroid or MMF withdrawal are fewer infections, low osteoporosis, and a possible improved cardiovascular risk profile, mainly due to lower levels of diabetes mellitus.

The introduction of CNIs, first cyclosporine and then tacrolimus, has greatly reduced the number of acute rejections in the first year and improved graft survival. However, there are some obvious downsides to the use of CNI, such as nephrotoxicity, hypertension, DM, and the increased risk for malignancy (117). The latter is dose-dependent, and particularly, relatively high exposition to CNI increases these risks (118–121). A number of studies have attempted to discontinue or lower tacrolimus in patients considered to be at low risk for TCMR (e.g., using ELISPOT-defined frequencies of interferon-gamma producing alloreactive T cells), but all showed an increase in TCMR (122, 123). This underscores the pivotal role of CNI in TCMR prevention, but it should be noted that most of these studies were performed relatively shortly after transplantation. However, attempts to wean recipients off tacrolimus at least 4 years posttransplant were discontinued because of either rejection or the formation of *de novo* DSA (124).

Given these results, CNI is usually continued for an indefinite period of time with the potential risk of nephrotoxicity. However, graft failure due to chronic interstitial fibrosis with tubular atrophy comprised only 5% of all graft losses in a single center cohort of over 700 patients with at least 15 years of follow-up (8). Although, in part, this may represent ongoing TCMR (iIFTA) or activity of non-HLA antibodies (61), it seems likely that continuous exposure to CNI may be the cause in a number of patients. In conclusion, CNI is the cornerstone of immune-suppressive regimens, and withdrawal at any point after transplantation increases the risk for TCMR and/or DSA formation. Therefore, the safest option is to continue but minimize CNI exposure in the long run.

Over the last decade, it has become clear that long-term kidney graft loss other than death with a functioning graft is dominated by chronic antibody-mediated rejection (8, 14). Although microvascular inflammation with signs of chronic endothelial activation (manifested by membrane multilayering) is invariably found, evidence of antibody-mediated rejection, such as complement C4d deposition in the kidney or the presence of circulating DSA, can be missing in up to 40% of cases (125–127). This discrepancy could be due to a lack of sensitivity of the used assays, as both DSA-negative and positive c-aABMR have similar histology, gene expression profiles, and graft survival (127, 128). However, in a small and specific subset of DSA-negative cases, other rejection mechanisms, such as those involving NK cells, could potentially provide an explanation (129).

Studies on graft survival have shown that tacrolimus trough levels below 5 ug/L are associated with decreased graft survival (19). However, this relationship between tacrolimus trough levels and graft survival is also dependent on HLA epitope mismatch load, as both are associated with the risk of TCMR and ABMR (84, 85, 130). It is important to realize that, even with adequate trough levels of tacrolimus (between 6 and 7 ug/ml), chronic antibody-mediated rejection cannot be fully prevented (131). It is not known what the contribution of MMF, prednisone, or combined tacrolimus use is to the prevention of c-aABMR. The recognition that c-aABMR is one of the major causes of death-censored graft loss has diminished the enthusiasm for immune suppression reduction protocols and research in this area. As no proven effective treatment is known for c-aABMR to date, the consensus is “optimization of maintenance immune suppressive medication” (132), which is translated into reinstitution of prednisone and/or higher tacrolimus levels in many instances. Whether this optimization will halt further deterioration of allograft function is questionable, as the underlying immunological process is an established coordinated T- and B-cell response to immunogenic epitopes derived from continuously exposed proteins.

### Biomarkers predict rejection and monitor alloreactivity against the donor organ

Minimizing the number of HLA mismatches, or HLA-epitope mismatch load, is the most straightforward strategy for reducing the risk for both TCMR and ABMR in the early and late periods after transplantation (133). Pre-transplantation DSA, also non-complement binding and only detectable in the Luminex assay, constitutes an increased risk for ABMR early and even many years after transplantation, leading to an estimated excessive graft loss of approximately 10–15% at 10 years (134, 135). Why the majority of DSA are not causing clinically relevant ABMR and/or graft loss is not well known but may relate to the affinity, complement binding capacity, and glycosylation profile of the antibodies (136–138). The current knowledge is insufficient to accurately predict which recipient with, e.g., a fully HLA-mismatched kidney transplant, will experience rejection and who will not.

The ELISPOT assay to determine the frequency of interferon-gamma (IFN)-producing alloreactive T cells in circulation is the most thoroughly investigated assay in relation to the risk of early acute rejection. Although a high frequency of IFN-producing T cells is associated with a higher risk of early acute rejection, the positive predictive value is rather poor (139). Lowering immune suppression early after transplantation in recipients with a low IFN-ELIspot count relative to donor HLA increased the risk of acute rejection (123). Currently, no biomarkers are available for adequate prediction of the long-term risk of rejection, but regular monitoring for *de novo* DSA can identify recipients who have developed DSA and in whom immune suppression should not be lowered.

**Figure 4:**
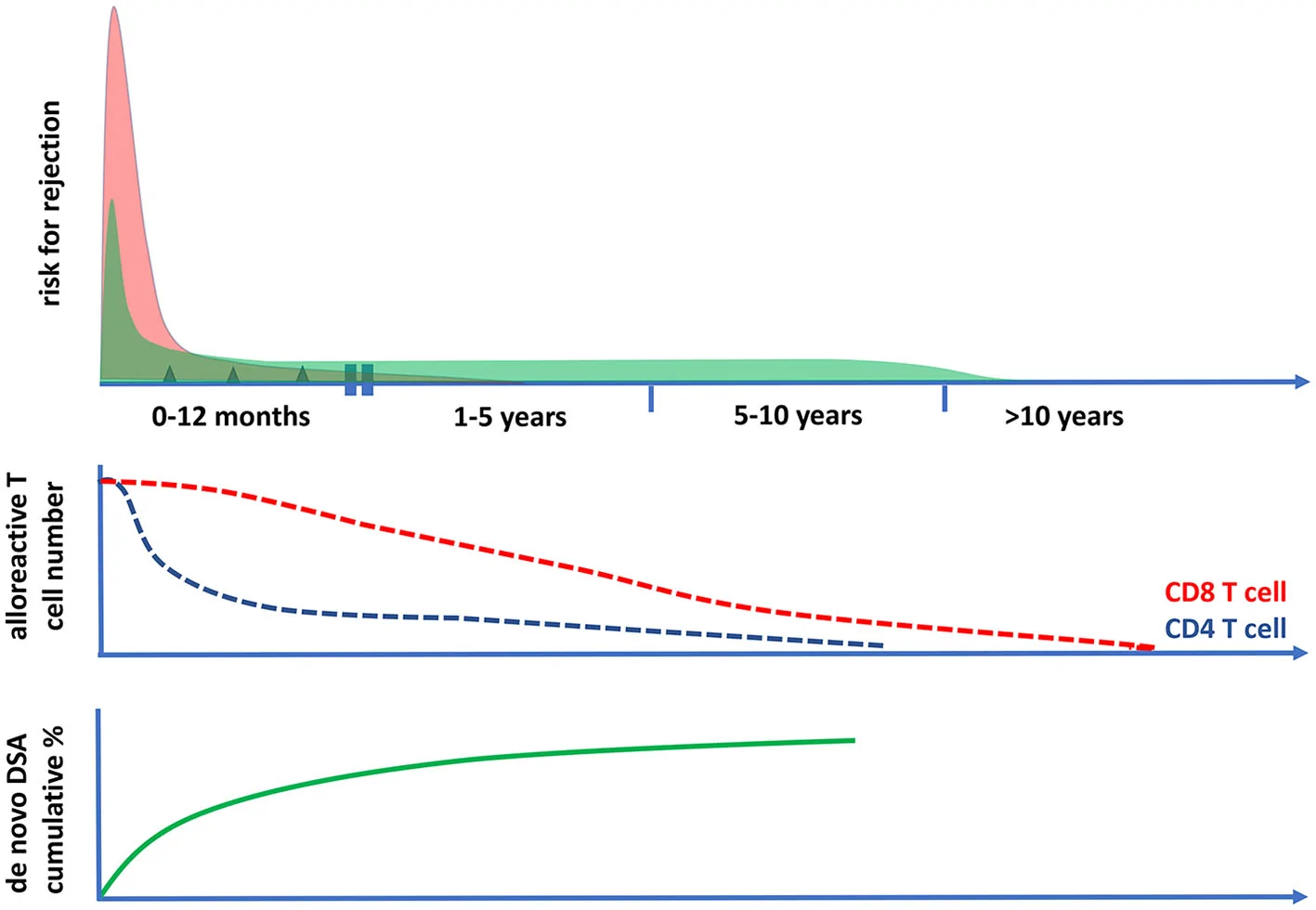
The risk for cellular and antibody-mediated rejection in time after transplantation in relation to the frequencies of circulating polyfunctional alloreactive T cells. The risk of T cell-mediated rejection (the upper panel and the red curve) after kidney transplantation is highest in the first month after transplantation; it then becomes low after 1 year and rare after 5 years post-transplantation. This coincides with the decrease in time of circulating polyfunctional CD4 T cells with direct alloreactivity (middle panel), while alloreactive CD8 T cell numbers become very low only after 10 years post-transplantation. The risk for antibody-mediated rejection (ABMR) can be initially increased post-transplantation (upper panel, green curve), which is largely determined by the presence of preformed donor-specific antibodies (DSA) at the time of transplantation. Thereafter, there is a fairly constant incidence of ABMR, which is associated with the cumulative increase of de novo DSA plateauing within 5–10 years after transplantation (lower panel). The incidence of clinical ABMR becomes very low beyond 10 years post-transplantation.

In conclusion, the intensity of immune suppression needed to prevent rejection-related graft loss changes over time (Figures 3, 4). Early after transplantation, high, intense immune suppression is needed to control high-risk direct alloreactive memory T cells, which decrease gradually, allowing for dose reduction over time. Maintenance immune suppression is then needed to control both persisting levels of low-risk direct alloreactivity and ongoing indirect alloreactivity. In the very long term (>10–15 years), even lower doses are likely sufficient to control indirect alloreactivity.

## Managing maintenance immune suppression regimens in relation to age and taking care of an older recipient

The age of the recipient has important implications for the management of immune suppressive drugs, as age impacts the risk of rejection, infection, and side effects. For instance, early steroid withdrawal especially benefits older adults in terms of posttransplant diabetes mellitus prevention. Ahn et al. have demonstrated in USRDS data in over 12,000 kidney transplant recipients that recipients over 55 years had a significantly higher benefit of steroid withdrawal than younger recipients (aHR for diabetes: 0.71 for >55 years vs. 1.18 in the age category 18–29 years) (107). Furthermore, a major competitive risk factor for graft loss in older adults is death with a functioning graft.

The aged immune system makes the older population more prone to infectious complications (140, 141), which explains the reverse relation between the risk of TCMR and age, an association that is continuous throughout adulthood (8, 97). For instance, recipients over 55 years of age at the time of transplantation have a twofold reduced risk for TCMR compared to recipients aged 18–45 years, (7, 8) but an increased risk for death from especially bacterial infections (142, 143). The number of HLA mismatches and pre-transplantation anti-HLA DSA are risk factors for ABMR in all age groups, including older adults (8, 68). Based on studies on *for-cause* kidney biopsies, time to c-aABMR diagnosis usually ranges between 4–6 years after transplantation, with poor graft survival leading on average to 50% graft loss 3 years after c-aABMR diagnosis (144, 145). However, as death with a functioning graft is the leading cause of graft loss in the majority of recipients over 55 years of age, mortality constitutes a significant competitive risk factor for c-aABMR as the cause of late graft failure.

Based on these data, it was postulated that patients with a low risk for rejection (<4 HLA MM, no positive PRA or DSA pre-transplantation) could benefit from tacrolimus monotherapy at 1-year post-transplantation without increasing the risk for rejection-related graft loss. An RCT based on this concept was recently published with 5-year follow-up data and indeed showed that this may be a feasible approach as the results showed no development of DSA, no increased rejection rate, significantly fewer infections, and a much better serological response after COVID vaccination (112, 146). Tacrolimus was given as a once-daily formulation, increasing medication adherence, and there were fewer gastrointestinal symptoms after MMF discontinuation (83, 113, 147). Of note, the mean age of the recipients in this trial was 59 years, and recipients with a TCMR treated with T cell-depleting therapy were excluded from randomization. Therefore, the results showed that, for monotherapy with a slow-release tacrolimus preparation 1 year after transplantation in older adults, immunologically standard-risk recipients is safe and associated with fewer gastrointestinal side effects and infections. As no DSA development was observed over a period of 4 years, the likelihood of c-aABMR development is deemed low.

Whether similar results can be obtained in young immunologically standard-risk recipients is not known, and death is not a major cause of graft failure in this age category. However, additional steering of immunosuppressive therapy by virus-specific T cell levels showed that, in pediatric patients, the load of immune suppressive medication could be lowered (148). In the age group of 19–45 years, the risk for acute TCMR also becomes virtually zero after 5 years, and the risk for chronic humoral rejection appears not to be influenced by age. Particularly in this group of recipients, the cumulative exposure to immune suppressive drugs may be substantial, as mortality is low and the expected transplant life, particularly with a living donor kidney transplant, is very good, at least 15 years. Therefore, although the reasons for lowering immune suppressive drug doses may differ with recipients' age, strategies to lower IS drug doses in the long run after transplantation can be considered an unmet need in all age categories of recipients.

### Alternative immune suppressive drugs

Several strategies have been attempted to reduce or withdraw CNI use in the long term, primarily to prevent the potential nephrotoxicity of CNI, by a switch to or combination with belatacept (a fusion protein blocking the costimulatory molecule CTLA-4) or an mTOR inhibitor. *De novo* use of belatacept is associated with an increased risk for rejection, but a switch from CNI to belatacept several years after transplantation may be a safe choice in selected patients who show progressive loss of transplant function because of CNI nephrotoxicity (149). However, belatacept increases the risk of infection and lowers vaccination responses and may thus not be ideal for older recipients, although specific clinical data for this age group are lacking (150). Using mTOR inhibitors in combination with CNI may allow for lower trough levels of CNI, and the effect on long-term graft survival and DSA formation at 2 years was comparable (151). Recently, an RCT has started in elderly (>65 years) kidney transplant patients comparing the standard maintenance IS regimen of tacrolimus/MMF/prednisone with low-dose tacrolimus/everolimus/prednisone (152). However, the primary outcome is graft function at 1 year, not patient survival, infections, or late rejection. The effect of different immune suppressive drugs on AICD of alloreactive T cells and the appearance of DSH after transplantation is essentially unknown.

### The benefits of a living donor kidney vs. a deceased donor kidney in older adults

A kidney from a living donor has some major advantages for an older recipient. Avoiding an inflamed allograft due to ischemic reperfusion injury lowers the risk of acute rejection, with delayed graft function being a rarer event and, on average, having a better graft function. Currently, the frequency of transplantation with a living donor kidney is much lower in older adults and is an underused option (153, 154). Data indicate that the kidneys of an older donor show almost similar excellent graft survival as the kidneys of a young donor (155). In addition, kidney donation by an older living donor in good clinical condition can be safely performed, even by an 85-year-old, with excellent graft function for many years (156, 157). Therefore, promoting living kidney donation in older adults appears to be an attractive strategy for improving the results of transplantation in older adults. The life-time risk for end-stage disease may be even lower in an older donor, and less strict rules for a donation could be acceptable, both given the diminished life span, e.g., a potential donor over 70 years of age. Currently, these deliberations are largely hypothetical as there is a lack of data on this subject, but they are of considerable interest for further exploration.

The Eurotransplant Senior program (old-for-old program) is designed to reduce waitlist time in older recipients by allocating preferentially kidneys of deceased donors from 65 years or older to recipients of 65 years or older without considering the degree of HLA matching. In particular, with the acceptance of kidneys from older donors after circulatory death, the frequency of DGF is high, and 64% do not reach an eGFR above 30 ml/min (6). Patient survival in the old-for-old group is similar compared to patients remaining on the waitlist, and mental and physical performance improve after transplantation (6, 158). However, results can be much better with a kidney from a living donor and are more cost-effective (155, 159). Moreover, given the lower risk of acute rejection, the risk of morbidity associated with anti-rejection treatment is lower, and an opportunity for less intense immune suppression exists.

## Future perspectives

The key differences in strategy for older adults compared to younger recipients are the concept of an age-related decrease in TCMR (but probably not ABMR), an increase in infection-related mortality and posttransplant DM (thus a benefit of steroid and MMF withdrawal), and increased mortality as a major competitive risk factor (decreasing the risk for c-aABMR-related graft loss substantially).

As recommendations for further studies in older adults, early steroid withdrawal is feasible and beneficial, while early tacrolimus withdrawal or lowering is associated with a substantial risk of acute rejection and should not be part of a protocol. The immunological standard-risk patients that have not experienced severe acute rejection (thus have clinical evidence of low immunological risk) are likely the most suitable group of recipients for a switch to, e.g., tacrolimus monotherapy after at least a year and maybe even at a low dose (trough levels 5 or less), e.g., 10 years after transplantation, making use of the development of profound DSH and the plateauing of newly diagnosed c-aABMR.

A major hurdle to answering these questions in an RCT is the very large number of recipients needed for inclusion and to be followed for at least 5–10 years when chronic rejection is taken as the primary endpoint. However, when infectious episodes, particularly in an older patient, are taken as the primary endpoint, a significant difference between groups has already been reached, with approximately 40 recipients per group and a follow-up of 5 years (146). It seems likely that such a difference in the infection rate will also lead to a difference in infection-related mortality in older transplant recipients.

In summary, the majority of kidney transplant recipients have excellent graft survival in the first year, given the efficacy of current maintenance immune suppression and effective treatment of acute rejections. The development of donor-specific hyporesponsiveness and the time-dependent formation of DSA, which tend to plateau after 10 years, provide an opportunity to reduce the intensity of immune maintenance suppression, particularly in older recipients. Given the high burden of infection and risk of death with a functioning graft in this age group, an RCT in the older transplant population is warranted with clinical endpoints other than chronic rejection-related graft loss.

## Author contributions

MB and AD contributed equally to the design and writing of the manuscript. Both authors contributed to the article and approved the submitted version.
