# Correction: Lowering maintenance immune suppression in elderly kidney transplant recipients; connecting the immunological and clinical dots

**DOI:** 10.3389/fmed.2026.2002921

**Published:** 2026-10-28

**Authors:** Michiel G. H. Betjes, Annelies De Weerd

**Affiliations:** Department of Internal Medicine, Erasmus MC Transplant Institute, University Medical Center Rotterdam, Rotterdam, Netherlands

**Keywords:** aging, immunity, immunosupperssion, kidney, transplantation T cell-mediated rejection, kidney transplantation, graft survival, age, mortality, antibody-mediated rejection, donor-specific antibodies, immune suppression

There was a mistake in the caption of Figure 2 as published. The description of the figure erroneously states that recipient APCs take MHC from “recipient cells”. The corrected caption of Figure 2 appears below:

“Recognition of allogeneic MHC by the recipients’ T cells and the routes to cellular and antibody-mediated rejection. T cells can be activated via their T cell receptor by allogeneic MHC molecules (MHC-TCR) expressed on antigen-presenting cells (APC). Co-stimulation by, for example, CD28-CD80 interaction is an important second signal needed for full T-cell activation and proliferation. Direct recognition is the activation of the recipient T cell by the donor APC. Recipient APC within the transplanted kidney can take MHC molecules from donor cells and insert them into their cell membrane (“crossdressing”). Through this mechanism, the recipient APC can present allogeneic MHC to recipient T cells, which is called semi-direct recognition. Both CD4 and CD8T cells can be activated by the direct and semi-direct pathways and cause acute T cell-mediated rejection, as indicated by the lightning arrow. Under the influence of immune suppressive (IS) medication, the activated T cells cannot be fully activated and may undergo apoptosis via a process called activation-induced cell death, which, in time, reduces the number of alloreactive T cells. The indirect pathway of recognition involves the classical route of antigen (e.g., donor MHC) taken up by the recipient APC and, after processing into an immunogenic peptide, presented in the context of MHC II to the recipient CD4T cells. Antigen-specific CD4T cells can interact with B cells to facilitate the transition to plasma cells producing donor-specific antibodies (DSA), a process that needs co-stimulation by CD40-CD40L interaction. DSA can cause antibody-mediated rejection, as indicated by the lightning arrow.”

The original version of this article has been updated.

